# Tripartite Motif-Containing Protein 65 (TRIM65) Inhibits Hepatitis B Virus Transcription

**DOI:** 10.3390/v16060890

**Published:** 2024-05-31

**Authors:** Sheng Shen, Ran Yan, Zhanglian Xie, Xiaoyang Yu, Hongyan Liang, Qiuhong You, Hu Zhang, Jinlin Hou, Xiaoyong Zhang, Yuanjie Liu, Jian Sun, Haitao Guo

**Affiliations:** 1 Department of Infectious Diseases, State Key Laboratory of Organ Failure Research, Key Laboratory of Infectious Diseases Research in South China, Ministry of Education, Guangdong Provincial Key Laboratory of Viral Hepatitis Research, Nanfang Hospital, Southern Medical University, Guangzhou 510515, China; shen5920@smu.edu.cn (S.S.); xiezhanglianny@foxmail.com (Z.X.); lianghongyan@smu.edu.cn (H.L.); youqiuhong38@smu.edu.cn (Q.Y.); jlhousmu@163.com (J.H.); xiaoyzhang@smu.edu.cn (X.Z.); 2 Department of Microbiology and Molecular Genetics; Cancer Virology Program, UPMC Hillman Cancer Center, University of Pittsburgh School of Medicine, Pittsburgh, PA 15213, USA; xyu4@andrew.cmu.edu (X.Y.); zhangh13@upmc.edu (H.Z.); liuy30@upmc.edu (Y.L.); 3 Department of Microbiology and Immunology, Indiana University School of Medicine, Indianapolis, IN 46202, USA; smileyanran@gmail.com

**Keywords:** HBV, tripartite motif proteins, TRIM65, HNF4α, transcription

## Abstract

Tripartite motif (TRIM) proteins, comprising a family of over 100 members with conserved motifs, exhibit diverse biological functions. Several TRIM proteins influence viral infections through direct antiviral mechanisms or by regulating host antiviral innate immune responses. To identify TRIM proteins modulating hepatitis B virus (HBV) replication, we assessed 45 human TRIMs in HBV-transfected HepG2 cells. Our study revealed that ectopic expression of 12 TRIM proteins significantly reduced HBV RNA and subsequent capsid-associated DNA levels. Notably, TRIM65 uniquely downregulated viral pregenomic (pg) RNA in an HBV-promoter-specific manner, suggesting a targeted antiviral effect. Mechanistically, TRIM65 inhibited HBV replication primarily at the transcriptional level via its E3 ubiquitin ligase activity and intact B-box domain. Though HNF4α emerged as a potential TRIM65 substrate, disrupting its binding site on the HBV genome did not completely abolish TRIM65’s antiviral effect. In addition, neither HBx expression nor cellular MAVS signaling was essential to TRIM65-mediated regulation of HBV transcription. Furthermore, CRISPR-mediated knock-out of TRIM65 in the HepG2-NTCP cells boosted HBV infection, validating its endogenous role. These findings underscore TRIM proteins’ capacity to inhibit HBV transcription and highlight TRIM65’s pivotal role in this process.

## 1. Introduction

Hepatitis B virus (HBV) belongs to the *Hepadnaviridae* family, a group of DNA viruses that replicate their genome by utilizing reverse transcription of a viral RNA template within hepatocytes [1]. Upon virus infection, mediated by a hepatocyte-specific viral receptor called sodium taurocholate co-transporting polypeptide (NTCP) [2], the 3.2 kb relaxed circular (rc) virion DNA is transported to the nucleus and converted into a nucleosome-decorated covalently closed circular (ccc) DNA mini-chromosome [3]. Using the cccDNA as the transcription template, five viral mRNAs are transcribed, including 3.5 kb precore and pregenomic (pg) RNA, 2.4 and 2.1 kb surface (envelope) mRNA, and 0.7 kb X mRNA [4]. HBV employs the host mRNA transcription machinery for its own transcription process, and the engagement of hepatocyte-enriched transcription factors represents the secondary mechanism underlying the virus’s hepatotropism [5]. The pgRNA serves not only as the template for the translation of the viral core protein and polymerase (pol) but also for reverse transcription. The pol/pgRNA complex is encapsidated by the core proteins into a nucleocapsid, inside of which the reverse transcription occurs to synthesize the progeny viral rcDNA [6,7]. Subsequently, mature nucleocapsids are enveloped by viral envelope proteins and secreted as progeny virions. Alternatively, the newly synthesized rcDNA can be redirected into the nucleus to replenish the cccDNA pool, thereby maintaining a persistent state of HBV infection [1,8].

The dynamics of HBV infection hinges on the delicate interplay between viral replication and host functions. As an obligate pathogen, HBV highly relies on the hepatic cellular machinery to complete its life cycle, but on the other hand, certain intrinsic and extrinsic host factors also restrict the replication of HBV. To date, a plethora of host restriction factors for HBV infection and reproduction have been identified under basal and/or cytokine-inducible expression conditions, representing an active area of research [5,9,10,11,12,13,14]. Therefore, it is of interest to identify additional novel host proteins that impede HBV replication at different steps of the viral life cycle, which will contribute to a comprehensive understanding of HBV–host interactions, paving the way for further mechanistic investigations and the development of potential host-targeting antiviral agents. 

Tripartite motif (TRIM)-containing proteins constitute a diverse family of over 100 RING domain E3 ubiquitin ligases [15]. TRIM proteins share a conserved N-terminal structure consisting of a RING finger domain, one or two B-boxes, a putative coiled-coil domain, and a variable C-terminus; the C-terminal variable region plays a crucial role in substrate recognition, thereby conferring functional specificity, and the PRY-SPRY domain is the most common C-terminal domain among known TRIM proteins [15,16]. TRIMs participate in a variety of cellular processes, including immunity, antiviral defense, and development [17,18,19]. Several TRIM proteins are interferon (IFN)-inducible and can restrict viral infections [20]. TRIM5α is one of the extensively studied TRIM proteins protective against the infection of various viruses, such as human immunodeficiency virus 1 (HIV-1) and poxviruses [21,22,23]. TRIM25 is critical to the cytoplasmic retinoic acid-inducible gene-I (RIG-I)-elicited antiviral innate immune response [24]. TRIM21 inhibits influenza A virus replication by degrading the viral matrix protein 1 (M1) [25]. As for the inhibition of HBV replication, it has been reported that TRIM22 inhibited HBV core promoter activity [26]; TRIM5γ, TRIM21, and TRIM31 promoted the ubiquitination of hepatitis B X protein (HBx) for proteasome degradation [27,28,29,30]; and TRM21 could also target HBV pol for proteasomal degradation [31]. A previous screen of 38 ectopically expressed TRIMs in HepG2 cells identified eight hits (TRIM5/6/11/14/25/26/31/41) capable of inhibiting viral transcription in an HBV transfection assay, but only TRIM41 exhibited some degree of HBV-promoter-specific inhibition in the reporter assay [32]. Nonetheless, with the large array of known TRIM proteins, it remains both intriguing and imperative to uncover novel TRIMs involved in regulating HBV replication, followed by in-depth mechanistic investigation. 

In this study, we systematically examined 45 TRIM proteins for their ability to inhibit HBV RNA transcription and/or DNA replication in HBV-transfected human hepatoma HepG2 cells. Among the 12 identified TRIM hits that inhibited HBV replication, primarily via reducing the steady state level of viral pgRNA, TRIM65 was selected for further validation and mechanistic study due to its potent and specific inhibitory effect on HBV transcription.

## 2. Materials and Methods

### 2.1. Cell Cultures and Drugs

HepG2 and Huh7 cells were maintained in Dulbecco’s modified Eagle’s medium (DMEM)/F-12 medium (Corning) supplemented with 10% fetal bovine serum, 100 U/mL penicillin, and 100 μg/mL streptomycin. HepDES19 cells were maintained in the same way as the HepG2 cells plus the addition of 1 μg/mL of tetracycline (tet) and 400 μg/mL of G418. To initiate HBV replication in the HepDES19 cells, tet was withdrawn from the culture medium, and the cells were cultured for the indicated time durations [33,34]. The HepG2-NTCP cell line, supporting HBV infection, has been described previously [35]. HepG2-shMAVS cells with stable knockdown of mitochondrial antiviral signaling protein (MAVS) were kindly provided by Dr. Zongdi Feng (the Research Institute at Nationwide Children’s Hospital) and cultured as previously described [36]. Recombinant human IFN-α2a, IFN-γ, and IFN-λ were purchased from PBL Biomedical Laboratories. MG132 was purchased from Sigma (#474790). Lamivudine (3TC) was a gift from Dr. William Mason (Fox Chase Center Center).

### 2.2. Plasmids and Transfection 

Plasmids expressing each individual TRIM protein listed in Table 1 were provided by Drs. Adolfo Garcia-Sastre (Mount Sinai Medical University), Andrea Ballabio (Telethon Institute of Genetics and Medicine), and Paul Bieniasz (Rockefeller University) [37,38,39]. The HBV replication-competent plasmids pHBV1.3 and pCMVHBV (both genotype D), for which the transcription of pgRNA is governed by the viral core promoter and the human cytomegalovirus immediate-early (CMV-IE) promoter, respectively, have been described previously [40]. The HBV replication plasmid pHBV-48 (genotype A) and its derivative pHBV-48-4A-Mut harboring HNF4α binding site mutations within the enhancer I region (nt 1134-TGAACCTTTACCC-nt 1146 to TCTACGGCTACCC) were provided by Dr. Pei-Jer Chen (National Taiwan University College of Medicine) [41,42]. Plasmid pHBV1.3ΔHBx (genotype D) was obtained from Addgene (#65461, a gift from Dr. Wang-Shick Ryu) [43]. Plasmids expressing N-terminally FLAG-tagged wild-type (WT), RING domain deletion (ΔR), RING and B-box domain deletion (ΔRB), SPRY domain deletion (ΔS), and coiled-coil and SPRY domain deletion (ΔCS) human TRIM65 proteins, respectively, were provided by Dr. Rongbin Zhou (University of Science and Technology of China) [44]. The plasmid pFlag-TRIM65ΔB, containing an internal deletion of the B-box domain, and the plasmid pFlag-TRIM65 C15A, bearing the E3-ligase-inactive mutant C15A in the RING finger domain of the TRIM65 gene, were constructed using the Q5 Site-Directed Mutagenesis Kit (NEB). The HBV promoter–luciferase reporter plasmids (EnII/Cp-Luc, S1p-Luc, S2p-Luc) were described previously [45]. The CMV promoter *Renilla*–luciferase reporter plasmid pRL-CMV was purchased from Promega (#E2261). The cells were transfected with the indicated plasmid(s) using Lipofectamine 3000 (Life Technologies) according to the manufacturer’s directions.

### 2.3. Reporter Assay 

HepG2 cells in 96-well plates were transfected with the promoter–reporter plasmids plus vectors expressing the genes of interest with Lipofectamine 3000. pRL-CMV was transfected as a control or co-transfected for normalization of the transfection efficiency. For each transfection, an empty control plasmid was added to ensure that each transfection received the same amount of total DNA (200 ng/well). Three days after transfection, the luciferase activities were measured using a dual luciferase assay kit (Promega) and the BioTek Synergy 2 Multi-Mode Reader. 

### 2.4. Establishment of TRIM65 Knock-Out Cell Lines

The TRIM65 knock-out cell line was generated through CRISPR-mediated genome editing of the TRIM65 gene loci. The single guide (sg) RNAs targeting the expressed region of the human TRIM65 gene were designed at http://www.e-crisp.org/E-CRISP. In addition to the general criteria for sgRNA design, the sgRNA sequence (5′-GTCCGGCAGAAGAGCTCCAG-3′) was designed to target the B-box domain of the ORF of TRIM65. The synthetic sgRNA oligo pairs were annealed and cloned into the BbsI-digested lentiCRISPRv2 control vector (Addgene# 52961, a gift from Dr. Feng Zhang). Lentivirus preparations were performed according to the protocols from Dr. Feng Zhang’s lab (genome-engineering.org). Briefly, each lentivector was co-transfected with the packaging plasmids psPAX2 and pMD2.G (Addgene # 12260 and 12259, respectively, gifts from Dr. Didier Trono) at a molar ratio of 4:3:1 into 293T cells using Lipofectamine 2000 (Invitrogen), and 48 h later, the media were collected, centrifuged at 1000× *g* for 10 min, and filtered through a 0.45 um filter, and the virus titers were determined using a lentiviral titration kit. Lentiviral transduction of the HepG2-NTCP cells and puromycin selection were performed to generate control and TRIM65 stable knock-out cell lines, namely HepG2-NTCP-control KO and HepG2-NTCP-TRIM65 KO. The TRIM65 knock-out phenotype was confirmed using Western blot and Sanger sequencing. 

### 2.5. HBVRNA/DNA Analyses 

Intracellular HBV RNA and DNA (cytoplasmic core DNA and Hirt DNA) were extracted and subjected to Northern blot and Southern blot, respectively, as described previously [46,47]. Hybridization signals were exposed to a phosphor imager screen and scanned using a Typhoon FLA 7000 imager. For quantitative PCR (qPCR) detection of the HBV cccDNA, the extracted Hirt DNA was heat-denatured, followed by Plasmid-safe ATP-dependent DNase (PSAD) (cat# E3101K, Epicentre) treatment to remove the ssDNA denatured from the rcDNA, according to our previous publications [48,49,50,51]. Then, the pre-cleaned Hirt DNA samples were subjected to qPCR detection of HBV cccDNA and cellular mitochondrial DNA, as described previously [50]. qRT-PCR detection of HBV precore mRNA and cellular GAPDH mRNA was conducted according to our previous publication [50]. 

### 2.6. Cellular mRNA RT-qPCR 

Total RNA was extracted from the cells using TRIzol and then digested with RQ1 RNase-free DNase I (Promega, # M6101), followed by purification with an RNA Clean & Concentrator kit (Zymo Research). A total of 1 μg of RNA was reverse-transcribed into complementary DNA (cDNA) using the Transcriptor First Strand cDNA Synthesis Kit (Roche), and LightCycler 480 SYBR Green I Master (Roche) was used to amplify the cDNA according to the manufacturer’s directions. qPCR of selected transcription factor genes was performed using a LightCycler 480 instrument (Roche), with β-actin serving as the housekeeping gene control. All the primers are as follows: human HNF4α (forward: 5′-GGTGTCCATACGCATCCTTGAC-3′; reverse 5′-AGCCGCTTGATCTTCCCTGGAT-3′), human PPARα (forward: 5′-TCGGCGAGGATAGTTCTGGAAG-3′; reverse: 5′-GACCACAGGATAAGTCACCGAG-3′), C/EBPα (forward: 5′-AGGAGGATGAAGCCAAGCAGCT-3′; reverse: 5′-AGTGCGCGATCTGGAACTGCAG-3′), and β-actin (forward: 5′-TGGGCATGGGTCAGAAGGAT-3′; reverse: 5′-TCCATCACGATGCCAGTGGT-3′). The qPCR was performed under the following conditions: 10 s at 95 °C, 30 s at 60 °C, and 5 s at 72 °C, for 50 cycles. 

### 2.7. HBV Infection and HBcAg Immunofluorescence 

HBV virion particles were collected from the supernatant of the induced HepDE19 cells, and the virus genome equivalents (vge) were calculated as described previously [35]. The HepG2-NTCP cells were infected with HBV at 100 vge/cell following the procedure in our previous publication [52]. Six days post-infection, the cells were fixed and subjected to DAPI staining and HBcAg immunofluorescence, as described previously [35,52].

### 2.8. Western Blot Assay 

Whole cell lysate samples prepared using Laemmli buffer was resolved in a 4–12% gradient SDS-PAGE, and the proteins were transferred onto an Immobilon PVDF-FL membrane (Millipore). The membranes were blocked with WesternBreeze blocking buffer (Life Technologies) and probed with antibodies against FLAG-tag (SAB4301135, Sigma), TRIM65 (HPA021578, Sigma), MAVS (24930, Cell Signaling), β-actin (sc-8432, Santa Cruz), and the HBV precore/core [53]. The bound antibodies were revealed using IRDye secondary antibodies. The immunoblot signals were visualized and quantified using the LI-COR Odyssey system.

## 3. Results

### 3.1. Identification of TRIM Proteins That Inhibit HBV Replication

In our ongoing pursuit to identify host factors possessing antiviral effects against HBV replication, we assembled a library of 45 published TRIM protein expression plasmids collected from academic sources (Table 1) [37,38,39]. Our library comprises 26 TRIM proteins that overlap with those assessed for anti-HBV activity previously [26,29,31,32], while introducing 19 novel additions to our repertoire. To assess their effects on HBV replication, HepG2 cells were co-transfected with pHBV1.3 and a control empty vector or plasmids expressing individual TRIMs at a 1:1 ratio. Five days later, the cells were harvested, and the expression of each individual TRIM was confirmed by Western blot using an antibody against the HA epitope engineered at the N-terminus of the TRIMs; intracellular HBV RNA and DNA were analyzed using Northern and Southern blot hybridization, respectively. As summarized in Table 1, among the 45 TRIMs tested, the ectopic expression of 12 TRIMs (5δ, 10, 11, 15, 19, 21, 26, 47, 51, 58, 65, and 73) reduced both the HBV RNA and DNA by more than 40% compared to the control, but none exhibited significant inhibition of HBV DNA replication without affecting the viral RNA levels. While the antiviral properties of TRIM5, 11, 21, and 26 against HBV have been documented in prior research [29,31,32], the other eight hits represent novel candidates as anti-HBV TRIMs. However, it is worth noting that despite its reported anti-HBV activity [26], TRIM22 did not exhibit inhibition of HBV in our assay, a result consistent with the findings from a previous study [32]. 

Next, we examined whether the 45 TRIMs affected the levels of the HBV RNA transcribed from the pCMVHBV template. The results, in conjugation with the above pHBV1.3 results, demonstrated that while each TRIM either reduced HBV RNA or did not in both cases, TRIM65 was the only one causing a significant reduction in the HBV RNA transcribed from pHBV1.3 but not pCMVHBV (Table 1), indicating the viral-promoter-specific antiviral property of TRIM65. Due to its potent and selective inhibition of HBV transcription, we thus prioritized TRIM65 in further studies.

### 3.2. TRIM65 Inhibits HBV Replication Primarily via Downregulating Viral Transcription

**Figure 1:**
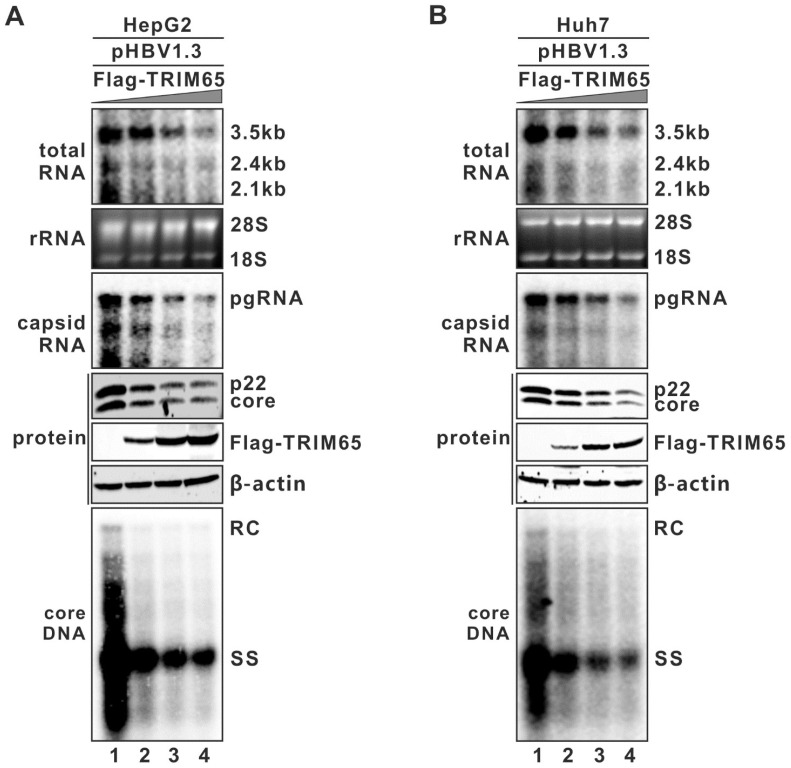
Ectopic expression of TRIM65 dose-dependently inhibits HBV replication primarily through reducing viral RNA. HepG2 (**A**) or Huh7 (**B**) cells seeded in 12-well plates were co-transfected with 0.6 μg of pHBV1.3 and an increasing amount of plasmid expressing FLAG-tagged TRIM65 (FLAG-TRIM65) (0, 0.3, 0.6, 1.2 μg). Control vector was supplemented to normalize the total number of transfected plasmids to 1.8 μg/transfection. Cells were harvested at day 5 post-transfection, and HBV total RNA and cytoplasmic encapsidated pgRNA were analyzed by Northern blot. The 28S and 18S ribosomal RNA (rRNA) served as total RNA loading control. HBV 3.5 kb precore (pC) mRNA and pgRNA and 2.4/2.1 kb surface mRNA are labeled. The lower-molecular-weight encapsidated pgRNA species represent the degradation intermediates catalyzed by the RNase activity of HBV pol during viral minus-strand DNA synthesis. HBV precore protein intermediate (p22) and core proteins and FLAG-tagged TRIM65 protein were detected by Western blot. HBV cytoplasmic core DNA was detected by Southern blot. RC, relaxed circular DNA; SS, single-stranded DNA.

To further determine the antiviral activity of TRIM65 against HBV replication, the FLAG-tagged wild type (WT) TRIM65 was co-transfected with pHBV1.3 into the HepG2 cells, followed by analyses of the intracellular viral total RNA and precore/core protein and core DNA. As shown in Figure 1A, the result clearly demonstrated that the ectopically expressed TRIM65 dose-dependently inhibited HBV replication primarily by reducing the levels of 3.5 kb viral RNA. A similar antiviral effect of TRIM65 on HBV was also observed in the pHBV1.3-transfected Huh7 cells (Figure 1B). 

**Figure 2:**
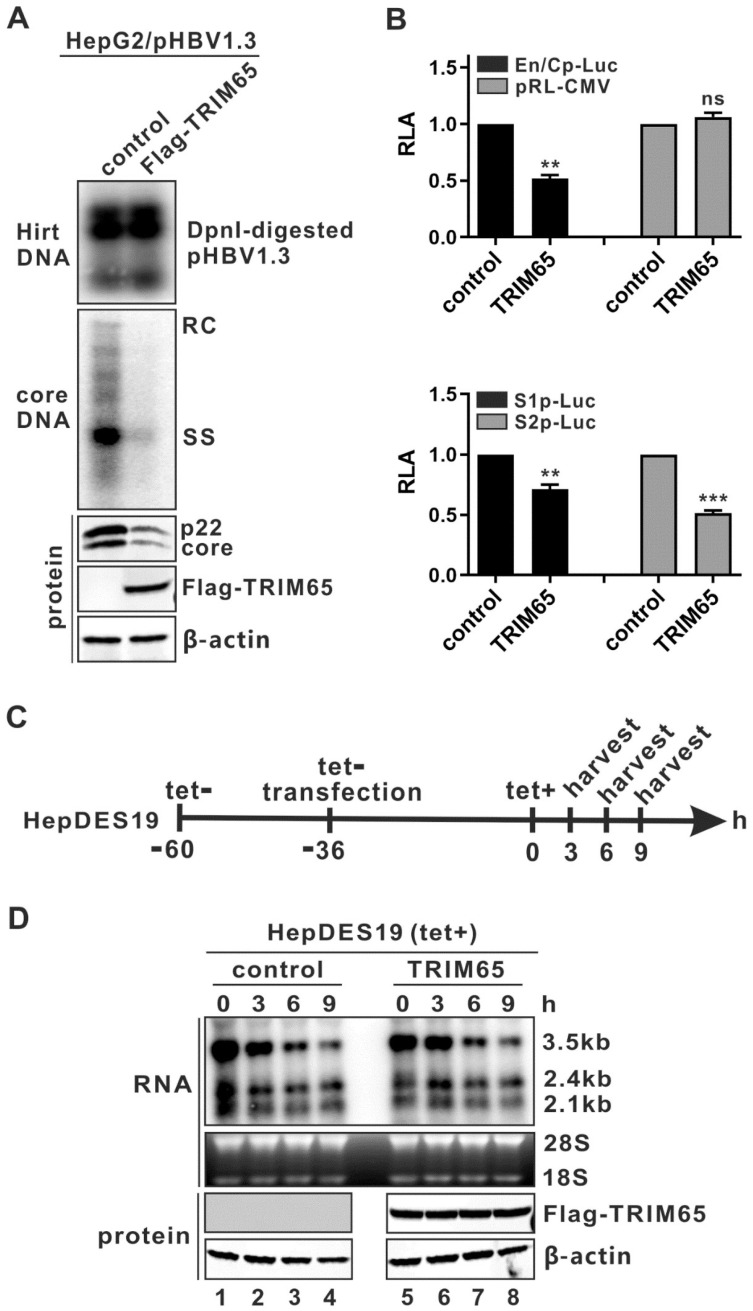
TRIM65 inhibits HBV replication at viral transcriptional level. (**A**) TRIM65 does not reduce the levels of transfected pHBV1.3 transcription template. HepG2 cells in 35 mm dishes were co-transfected with 2 µg of plasmid pHBV1.3 and 2 µg of control vector or plasmid FLAG-TRIM65. The cells were harvested on day 5 post-transfection and were subjected to Hirt DNA extraction. The Hirt DNA was then incubated with restriction enzyme Dpn I to digest the bacteria-derived plasmid DNA with Dam methylation. The Dpn I-digested Hirt DNA samples and the cytoplasmic HBV core DNA were analyzed by Southern blot. Viral p22 and core proteins, FLAG-tagged TRIM65, and β-actin were analyzed by Western blot. (**B**) TRIM65 inhibits HBV promoter activity. HepG2 cells seeded in a 96-well plate were co-transfected with 100 ng of EnII/Cp-Luc and 4 ng of pRL-CMV, plus 100 ng of control vector or plasmid FLAG-TRIM65. In separate wells, HepG2 cells were transfected with 100 ng of S1p-Luc or S2p-Luc, together with 100 ng of control vector or FLAG-TRIM65, with 4 ng of pRL-CMV also included in each transfection for normalization of transfection efficiency. Three days after transfection, cells were harvested, and luciferase activities were measured. The plotted relative luciferase activity (RLA) represents the mean ± standard deviation (SD, n = 3) of the ratios of absorbance obtained from wells expressing FLAG-TRIM65 over that obtained from wells that were transfected with control vector. ** *p* < 0.01; *** *p* < 0.001. (**C**) Schematic illustration of experimental design. HepDES19 cells were seeded in a 35 mm dish and cultured with tet-free medium to induce HBV RNA expression. Then, 24 h later, cells were transfected with 4 µg of control vector or plasmid FLAG-TRIM65 for 36 h; tet was added back to the culture medium to shut down pgRNA transcription; and cells were harvested at indicated time points. (**D**) TRIM65 does not promote HBV RNA decay in cell cultures. HBV RNA was extracted from harvested samples in (**C**) and analyzed by Northern blot. The expression of FLAG-TRIM65 was detected by Western blot, while β-actin served as loading control.

Considering that HBV plasmid DNA was the transcription template for viral RNA production in the transfection system, to determine whether TRIM65-mediated downregulation of the 3.5 kb viral RNA was due to a transcriptional or post-transcriptional mechanism, we first ruled out the possibility that TRIM65 may cause the degradation of transfected HBV plasmids. The input HBV plasmid DNA was recovered from the transfected cells using Hirt DNA extraction, digested by the bacterial DNA-methylation-specific restriction enzyme Dpn I, and subjected to Southern blot analysis, as previously described by us [45]. As shown in Figure 2A, the levels of pHBV1.3 plasmid DNA in the transfected cells were unchanged in the absence or presence of TRIM65 overexpression, as revealed by the Dpn I-digested fragments; however, consistent with the results in Figure 1, the viral precore/core protein and cytoplasmic core DNA were significantly reduced by the overexpression of TRIM65. 

Next, we assessed the potential effect of TRIM65 on viral promoter activity. The luciferase reporter assay demonstrated that TRIM65 overexpression significantly reduced the activities of the HBV enhancer II/core promoter (EnII/Cp), the S1 promoter, and the S2 promoter but caused no inhibition of CMV-IE promoter activity (Figure 2B), indicating that TRIM65 reduces 3.5 kb viral RNA transcription by inhibiting viral promoter activity. To further confirm this, we directly measured the decay kinetics of 3.5 kb viral RNA in the absence and presence of TRIM65 overexpression. For this purpose, the inducible HBV stable cell line HepDES19 was cultured in the absence of tet to induce HBV pgRNA transcription for 24 h, followed by transfection with the control vector or a TRIM65 expression vector for an additional 36 h. Tet was then added back to immediately shut down the de novo pgRNA transcription from the transgene, and the levels of the remaining intracellular HBV RNA were measured by Northern blot every 3 h for up to 9 h (Figure 2C). As shown in Figure 2D, the levels of HBV RNA at each time point were comparable between the control and TRIM65 overexpression groups, suggesting that TRIM65 does not promote HBV RNA degradation. Collectively, we conclude that TRIM65 inhibits HBV replication primarily through reducing viral pgRNA transcription.

### 3.3. TRIM65 Inhibits HBV Transcription Depending on Its E3 Ubiquitin Ligase Activity

**Figure 3:**
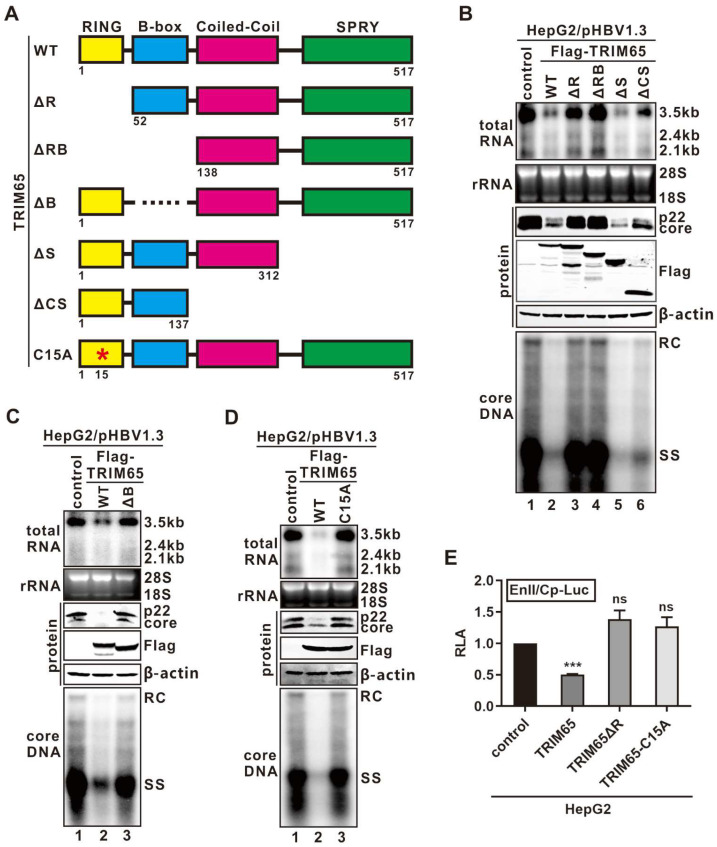
Mapping the functional antiviral domain of TRIM65. (**A**) Schematic structure of wild-type (WT) TRIM65 and mutants. The color rectangles indicate the known functional domains of TRIM65, specifically the RING finger (R), B-box (**B**), coiled-coil (**C**), and SPRY (S) domains. The amino acid (aa) positions are labeled with numbers. The E3 ligase enzymatically inactive mutant site (C15A) is marked with an asterisk. (**B**–**D**) The RING finger and B-box domains and E3 ligase activity of TRIM65 are indispensable for TRIM65’s antiviral effect. HepG2 cells were transfected with pHBV1.3 and equal amount of control vector or TRIM65 WT and (**B**) ΔR, ΔRB, ΔS, ΔCS; (**C**) ΔB; and (**D**) C15A. Cells were harvested 5 days post-transfection. Total RNA was subjected to HBV RNA Northern blot. 28S and 18S rRNA served as loading control. HBV p22/core proteins and FLAG-tagged TRIM65 proteins were detected by Western blot. HBV cytoplasmic core DNA was detected by Southern blot. (**E**) The RING finger domain and its E3 ligase activity are required for TRIM65-mediated inhibition of HBV promoter activity. HepG2 cells seeded in a 96-well plate were co-transfected with 100 ng of EnII/Cp-Luc, together with 100 ng of control vector or FLAG-TRIM65 WT, ΔR, or C15A; a total of 4 ng of pRL-CMV was included in each transfection for normalization of transfection efficiency. Luciferase activities were measured three days after transfection and plotted as RLA (mean ± SD, n = 3; *** *p* < 0.001, ns: not significant).

TRIM65 contains four conserved domains from the N- to the C-terminus, including a RING finger domain, a B-box domain, a coiled-coil domain, and a C-terminal SPRY domain (Figure 3A) [15,16]. To determine the functional domain(s) which is responsible for TRIM65’s antiviral activity against HBV transcription, a serial of TRIM65 mutants with deletion of each individual functional domain was constructed; in addition, a key cysteine residue 15 in the RING finger domain essential for E3 ubiquitin ligase activity was mutated into alanine, giving rise to the E3-inactive mutant TRIM65C15A [54,55] (Figure 3A). The WT TRIM65 and the mutants were individually co-transfected with pHBV1.3 into the HepG2 cells, and their effects on viral RNA and DNA were evaluated. As shown in Figure 3B,C, while the WT TRIM65 efficiently inhibited HBV transcription, deletion of the RING finger domain and/or B-box domain, but not the coiled-coil domain or SPRY domain, completely abolished TRIM65’s antiviral effect. Furthermore, the E3-inactive mutant C15A failed to inhibit HBV transcription or replication (Figure 3D), and both the RING finger domain deletion mutant ΔR and the E3-inactive mutant C15A lost their inhibitory effect on the HBV EnII/Cp promoter activity (Figure 3E). Collectively, the above results suggest that the adjacent RING finger domain and the B-box domain are required to maintain TRIM65’s anti-HBV property, with the E3 ligase activity encoded by the RING finger domain being essential. 

### 3.4. HBx and MAVS Are Not Involved in the TRIM65-Mediated Antiviral Effect

**Figure 4:**
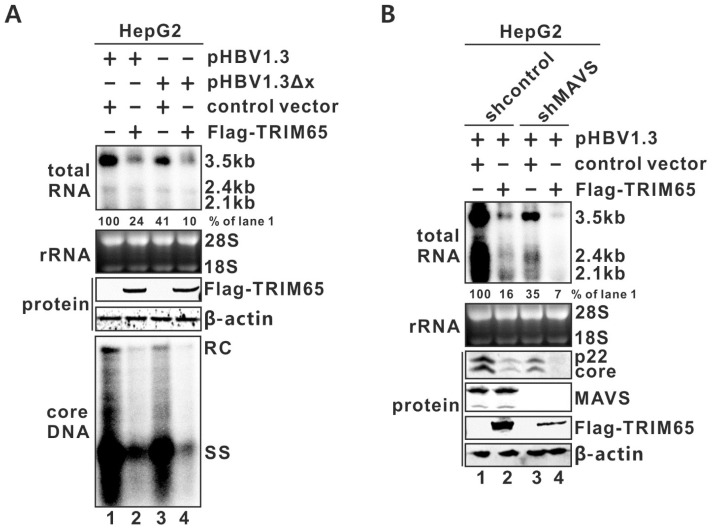
HBx or MAVS is not required for TRIM65-mediated inhibition of HBV transcription. (**A**) HepG2 cells were co-transfected with plasmid pHBV1.3 or pHBV1.3ΔX with either control vector or plasmid FLAG-TRIM65; (**B**) HepG2-shControl and -shMAVS cells were transfected with pHBV1.3 and equal amount of control vector or FLAG-TRIM65. Cells were harvested 5 days post-transfection. Total RNA was subjected to HBV RNA Northern blot. Cytoplasmic HBV core DNA was detected by Southern blot. HBV p22 and core proteins, cellular MAVS and β-actin proteins, and FLAG-TRIM65 protein were detected by Western blot.

HBx selectively transactivates HBV transcription from episomal DNA templates including the authentic cccDNA and the transfected HBV plasmid [50,56,57,58]. Previous studies have reported that several TRIMs (5γ, 21, 31) inhibit HBV transcription through interacting with HBx and promoting its degradation [27,28,29]. We thus assessed the dependency on HBx for the TRIM65-mediated antiviral effect in HepG2 cells transfected with TRIM65 plus pHBV1.3 or the HBx-null mutant pHBV1.3Δx. As shown in Figure 4A, the level of transcribed viral RNA was lower in the pHBV1.3Δx-transfected cells compared to that with pHBV1.3 transfection due to the loss of HBx (lane 3 vs. 1); however, TRIM65 remained functional in reducing HBV-plasmid-based viral RNA transcription and core DNA replication in the absence of HBx expression (lane 4 vs. 3), indicating an HBx-independent antiviral mechanism of TRIM65 (Figure 4A).

The cellular pattern recognition receptors (PRRs) RIG-I and MDA5, upon activation, utilize a signaling adaptor protein called mitochondrial antiviral signaling protein (MAVS) to activate NF-κB and the transcription factor interferon regulatory factor 3 (IRF3), which, in turn, induces type I IFN production [59,60]. It has been shown that TRIM65 specifically interacts with MDA5 and promotes the K63-linked ubiquitination of MDA5, which is critical for MDA5 oligomerization and activation [44]. Thus, we further tested whether MAVS is involved in the TRIM65-mediated inhibition of HBV transcription by comparing the antiviral effect of TRIM5 between the pHBV1.3-transfected HepG2-shMAVS and -shControl cells. As shown in Figure 4B, despite the undetectable level of MAVS in the HepG2-shMAVS cells, TRIM65 overexpression was still able to significantly suppress HBV RNA transcription (lane 4 vs. 3), indicating that MAVS is dispensable in the TRIM65-mediated anti-HBV mechanism.

### 3.5. TRIM65 Inhibits HBV Transcription Partially through Downregulating HNF4α 

**Figure 5:**
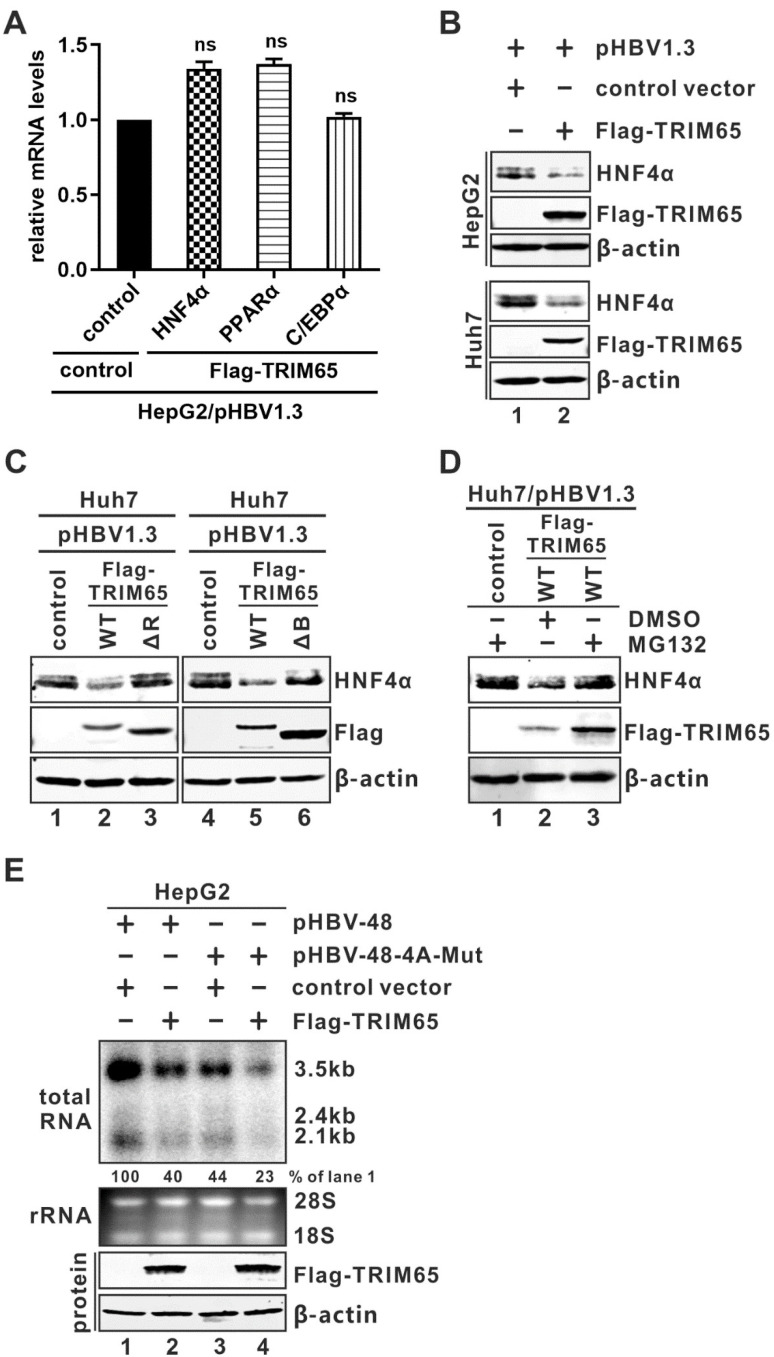
TRIM65 induces HNF4α downregulation. (**A**) Assessment of the effect of TRIM65 overexpression on mRNA levels of liver-enriched transcription factors (TFs). HepG2 cells were co-transfected with pHBV1.3 and control empty vector or FLAG-TRIM65 for 5 days; then, the mRNA levels of HNF4α, PPARα, and C/EBPα were analyzed by qRT-PCR and normalized by β-actin mRNA. The relative mRNA levels were plotted as fold change from control (mean ± SD, n = 3). (**B**) TRIM65 overexpression reduces HNF4α protein expression. HepG2 and Huh7 cells were co-transfected with pHBV1.3 and control empty vector or FLAG-TRIM65 for 5 days, followed by Western blot analysis of endogenously expressed HNF4α and overexpressed FLAG-TRIM65 proteins. β-actin served as loading control. (**C**) The RING finger and B-box domains are required for TRIM65-mediated HNF4α downregulation. Huh7 cells were co-transfected with pHBV1.3 and control empty vector or WT, ΔR, or ΔB FLAG-TRIM65 for 4 days, followed by Western blot analyses of HNF4α, FLAG-TRIM65 WT, and mutants. β-actin served as loading control. (**D**) TRIM65-mediated HNF4α reduction is rescued by treatment with proteasome inhibitor MG132. Huh7 cells were co-transfected with pHBV1.3 and control empty vector or WT FLAG-TRIM65. Four days later, the cells were treated with MG132 (20 µM) or solvent control DMSO as indicated for 24 h, followed by Western blot detection of HNF4α, FLAG-TRIM65, and β-actin. (**E**) Effect of TRIM65 on the transcription of HBV genome with HNF4α binding site mutation. HepG2 cells were co-transfected with plasmid pHBV48 or pHBV-48-4A-Mut with either control vector or FLAG-TRIM65. Cells were harvested 5 days post-transfection. Total RNA was subjected to HBV RNA Northern blot. FLAG-TRIM65 and β-actin proteins were detected by Western blot.

As a hepatotropic virus, HBV transcription requires liver-enriched transcription factors (TFs), including HNF4α, PPARα, and C/EBPα, which are crucial for cccDNA transcription [5,61,62]. To determine whether TRIM65 inhibits HBV transcription by affecting these TFs, pHBV1.3 was co-transfected with TRIM65 into HepG2 cells, and the mRNA levels of the above three TFs were analyzed by qRT-PCR. The results showed no reduction in the TFs due to TRIM65 (Figure 5A). Interestingly, we found that TRIM65 overexpression downregulated the HNF4α expression at the protein level (Figure 5B), but the TRIM65 RING finger and B-box domain deletion mutants did not exhibit this effect (Figure 5C). Furthermore, the wild-type TRIM65-mediated reduction in HNF4α was abrogated by treatment with the proteasome inhibitor MG132, even though TRIM65 was also expressed at a higher level under MG132 treatment (Figure 5D, lane 3 vs. 2). Collectively, the above evidence suggests that HNF4α is a plausible substrate for TRIM65-mediated ubiquitination and subsequent proteosome degradation. 

To further assess whether HNF4α is the major target of the TRIM65-mediated antiviral effect on HBV transcription, we employed an HBV construct pHBV-48-4A-Mut harboring HNF4α binding site mutations in the viral enhancer I region [41]. As shown in Figure 5E, pHBV-48-4A-Mut supported lower levels of HBV RNA transcription compared to the wild-type pHBV-48 due to the loss of HNF4a recruitment (lane 3 vs. 1); however, TRIM65 overexpression further reduced the level of HBV RNA transcribed from the pHBV-48-4A-Mut template (lane 4 vs. 3), inferring that HNF4α is unlikely the sole target of the TRIM65-mediated antiviral mechanism and additional TF(s) and mechanism(s) ought to be involved. 

### 3.6. The Antiviral Effect of Endogenous TRIM65 on HBV Infection

**Figure 6:**
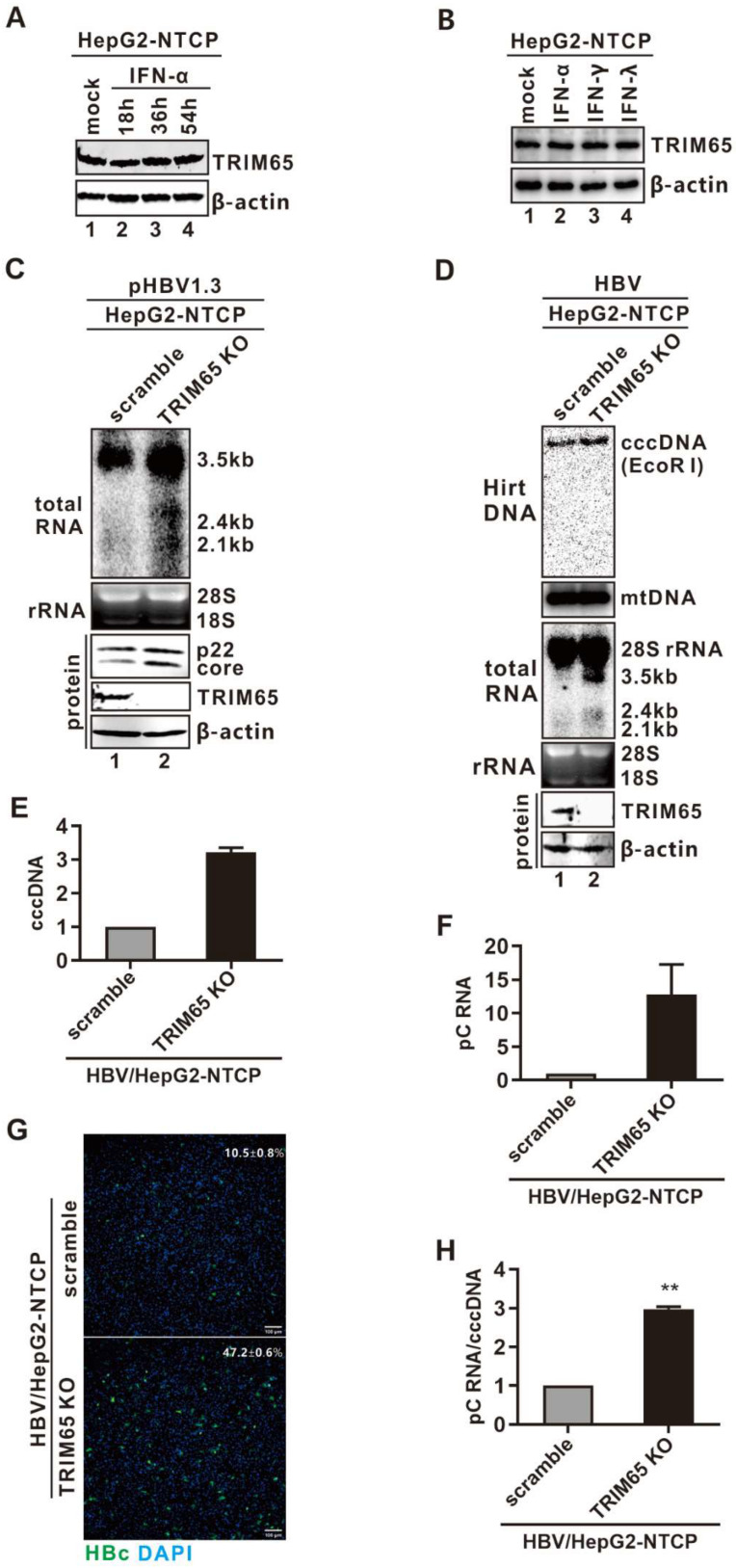
The effect of endogenous TRIM65 on HBV transcription. (**A**,**B**) Basal level of TRIM65 expression in hepatoma cells and its inducibility by IFNs. (**A**) HepG2-NTCP cells were left untreated or treated with human IFN-α (1000 IU/mL) for indicated time durations (18 h, 36 h, and 54 h). The untreated cells (control) were harvested together with the cells treated with IFN-α for 54 h. (**B**) Another group of HepG2 cells was left untreated (control) or treated with type I IFN-α (1000 IU/mL), type II IFN-γ (100 ng/mL), or type III IFN-λ (100 ng/mL) for 48 h (**B**). The levels of TRIM65 expression were determined by Western blot. β-actin expression was presented as loading control. (**C**) Knock-out (KO) of endogenous TRIM65 upregulates HBV transcription in virally transfected cells. HepG2-NTCP scramble control and TRIM65 KO cells were transfected with pHBV1.3 for 3 days, followed by Northen blot assay for HBV total RNA and Western blot analyses of HBV p22/core and cellular TRIM65 proteins. (**D**–**H**) KO of endogenous TRIM65 enhances HBV infection. HepG2-NTCP scramble control and TRIM65 KO cells were infected with HBV (100 vge/cell) for 6 days in the presence of 10 μM of 3TC, followed by the following assays: (**D**) total Hirt DNA was extracted, heat-denatured, and digested by EcoR I and subjected to Southern blot detection of the linearized cccDNA. Mitochondrial DNA (mtDNA) served as Southern blot loading control. Total RNA samples were subjected to HBV RNA Northern blot analysis. (**E**) An aliquot of the original Hirt DNA was heat-denatured and digested by PSAD to remove non-cccDNA species, followed by cccDNA qPCR quantification. The relative cccDNA levels were plotted as fold change compared to the scramble control group. (**F**) HBV pC mRNA in the total RNA samples was detected by qPCR. The relative pC mRNA levels were plotted as fold change compared to control group. (**G**) HBV cccDNA-based gene expression was assessed by HBc immunofluorescence; the average percentage of HBc-positive cells were calculated from five microscope fields of view. Nuclei were stained with DAPI. Scale bar: 100 μm. (**H**) The relative transcription activity of cccDNA was presented by normalizing pC mRNA level (**F**) to cccDNA level (**E**) and plotted as fold change compared to control group (mean ± SD, n = 3. ** *p* < 0.01).

Since several TRIMs are interferon-stimulated genes (ISGs) [18,20], we thus examined the basal level of TRIM65 in the HepG2-NTCP12 cells and its inducibility by IFNs. As shown in Figure 6A,B, the expression of endogenous TRIM65 was detected in the HepG2-NTCP12 cells by Western blot but could not be upregulated by any of the type I-III IFNs, indicating that TRIM65 is not an ISG. 

To study the antiviral effect of basal endogenous TRIM65 on HBV transcription, the endogenous TRIM65 in the HepG2-NTCP cells was completely depleted through gene knock-out by CRISPR/Cas9. Firstly, we compared the levels of HBV transcription and gene expression in pHBV1.3-transfected HepG2-NTCP scramble control knock-out (KO) and TRIM65 KO cells. As shown in Figure 6C, HBV RNA production and p22/core expression were markedly upregulated in the TRIM65 KO cells. Next, we assessed the antiviral effect of endogenous TRIM65 in HBV-infected HepG2-NTCP cells. Considering that HBV cccDNA is synthesized from both the rcDNA from incoming virions and the newly synthesized rcDNA during natural infection [8,63], to specifically study the effect of TRIM65 on the preexisting cccDNA transcription in the context of HBV infection, the HBV-infected HepG2-NTCP scramble KO and TRIM65 KO cells were treated with 3TC to block de novo HBV-replication-mediated cccDNA replenishment. It is known that nucleoside analog drugs do not block the cccDNA formation from incoming hepadnaviral virion DNA [64,65]. Upon HBV infection, the depletion of TRIM65 resulted in a marked upregulation of viral cccDNA and mRNA levels compared to those in the control group, as revealed by Southern and Northern blots (Figure 6D). In addition, there were significant increases in cccDNA (Figure 6E) and precore (pC) mRNA (Figure 6F), as demonstrated by the qPCR analysis, and a marked increase in intracellular HBcAg expression, as revealed by staining (Figure 6G), as compared to the controls. Since cccDNA replenishment via recycling of newly synthesized rcDNA was supposedly blocked by 3TC, the increased level of cccDNA in the TRIM65 KO cells was possibly due to unknown cell conditions favoring HBV entry, cccDNA formation, or stability. Nonetheless, after normalizing the qPCR values of the precore mRNA to cccDNA, the normalized cccDNA transcriptional activity remained significant in the TRIM65 KO cells compared to the control KO cells (Figure 6H), indicating a suppressive effect of endogenous TRIM65 on cccDNA transcription. In this regard, it is also plausible that the cccDNA stability is positively correlated with its transcriptional activity, as indicated in our recent study [51]. 

## 4. Discussion

Serving as the first line of host defense, the cellular innate immune system promptly detects viral infections and triggers the production of IFNs, which, in turn, induce ISGs to restrict viral replication and propagation [66,67]. Despite the fact that HBV has long been considered a stealth virus capable of evading innate activation, the virus shows susceptibility to IFN treatment and a handful of ISGs [14,68]. Many members of the TRIM family are ISGs and play important roles in host antiviral innate immunity [17,18,20]. In the present study, we identified 12 TRIM proteins that, upon ectopic expression, significantly reduced HBV reproduction, including viral mRNA and capsid DNA, in virally transfected HepG2 cells (Table 1). All 12 TRIM proteins appeared to inhibit HBV replication primarily at the viral transcriptional level. Among them, TRIM65 emerges as a novel anti-HBV TRIM exhibiting a viral-promoter-specific antiviral effect on HBV transcription (Figure 1 and Figure 2). It is worth noting that our TRIM screening assay was based on the ectopic expression of TRIM expression vectors collected from multiple sources (see Section 2); due to the different backbones of the plasmids, the protein expression levels of different TRIMs may vary, and the steady-state level of each TRIM protein also depends on its half-life. Furthermore, it is unknown whether the basal level of each TRIM influences the antiviral effect of its ectopically expressed one. In these regards, the screening results were not normalized by the expression levels of the TRIM proteins, and our main purpose was to select TRIMs of interest for further study based on their antiviral activity and HBV promoter specificity.

It has been reported that several other TRIMs, such as TRIM22 and TRIM41, inhibit HBV replication by regulating viral promoter activity [26,32]. TRIM22 is localized into the nucleus with the assistance of the C-terminal SPRY domain and inhibits HBV EnII/Cp promoter activity through the RING finger domain [26]. The C-terminal SPRY domain of TRIM proteins played a crucial role in substrate recognition in previous studies [16,18,19,38]. In mapping the signature TRIM domains responsible for the TRIM65-mediated antiviral effect, we found that the RING finger domain and its corresponding E3 ubiquitin ligase activity are essential for TRIM65 to inhibit HBV transcription (Figure 3). However, the C-terminal SPRY domain of TRIM65 is dispensable in this antiviral effect, indicating a distinct antiviral mechanism of TRIM65 against HBV. Moreover, the B-box domain is thought to enhance the substrate recognition and ubiquitination activities of TRIM5α and TRIM21 [69,70]. In line with this, the B-box domain of TRIM65 is essential in terms of its inhibitory effect on HBV transcription in our study (Figure 3). Thus, it is interesting to further investigate the role of the B-box domain of TRIM65 in substrate recognition and ubiquitination.

In search of potential substrate(s) targeted by TRIM65 to inhibit HBV transcription, our initial protein of interest was HBx, a key regulatory viral protein that stimulates and maintains HBV transcription from episomal templates, including the authentic cccDNA and its surrogates [50,56,57,58]. However, through comparative analyses of the antiviral effect of TRIM65 on the wild type and the HBx-null pHBV1.3 replicon, we found that HBx is not required for TRIM65-mediated inhibition of HBV transcription in a transfection system (Figure 4A). Notably, previous studies have identified at least six TRIM proteins, including TRIM5γ, TRIM14, TRIM21, TRIM25, TRIM26, and TRIM31, that induce HBx degradation in HBV-infected HepG2-NTCP cells during the early phase of infection, resulting in a restoration of the host restriction factor Smc5/6 complex to suppress cccDNA transcription [27,28,29,71,72,73]. While these studies await independent validation, including the Western blot detectability of HBx expression in HBV-infected HepG2-NTCP cells, it will also be intriguing to explore why the host cells deploy a variety of TRIM E3 ligases to deplete the same viral substrate and how HBV and HBx navigate the antiviral pressure exerted by multiple TRIMs to maintain a persistent infection. 

Next, we explored the possibility that MDA5 and its downstream signaling might be the target of TRIM65. MDA5 is a cytoplasmic PRR and can recognize viral dsRNA intermediates to activate type I interferon production after virus infection, thus possessing a critical role in antiviral innate immunity [59,74]. We have previously shown that the activation of MDA5 inhibits HBV replication in hepatoma cells, including HepG2 and Huh7 [47]. More intriguingly, a recent study demonstrated that TRIM65 specifically interacts with MDA5 and promotes the K63-linked ubiquitination of MDA5 to activate downstream MAVS signaling in HEK293T cells [44]. To test whether MAVS is involved in the TRIM65-mediated inhibition of HBV transcription, we employed a HepG2 cell line with stable knockdown of MAVS with shRNA [36]. However, the depletion of MAVS expression had no reversal effect on TRIM65’s antiviral activity (Figure 4B). Although we did not assess the reported interaction between TRIM65 and MDA5 or depleted MDA5 in the HepG2 cells used in our study, given that MAVS is the indispensable adaptor for MDA5 and RIG-I that activates the downstream antiviral innate signaling cascade, our results suggest that either MDA5 or MAVS is unlikely to be responsible for the TRIM65-mediated inhibition of HBV transcription. 

We have also taken an unbiased approach to looking for potential cellular factors involved in the TRIM65-mediated anti-HBV effect by using comparative transcriptomic analysis of pHBV1.3-transfected cells with and without TRIM65 overexpression. Among the TRIM65-induced differentially expressed genes (DEGs) with statistical significance, we did not see any gene of interest (GOI) that possesses a reported regulatory function in HBV transcription, such as a TF or epigenetic regulator. Interestingly, most of the TRIM65-induced DEGs are ISGs, which might be a consequence of the possible activation of MDA5 by TRIM65. However, the potential involvement of MAVS in the TRIM65-mediated antiviral effect was ruled out, as aforementioned. Furthermore, blocking the downstream signaling of MAVS by with dominant-negative IRF3 or NF-κB also failed to mitigate TRIM65’s anti-HBV activity, despite a complete abolishment of ISG induction. While further exploration of the TRIM65-induced transcriptomic changes is needed, alternative approaches should also be considered to further study the antiviral mechanism of TRIM65. 

Based on the result that TRIM65 specifically inhibits the activity of HBV authentic promoters (Figure 2), we thus hypothesized that TRIM65 may regulate liver-specific TFs. In the liver, HNF4α controls about one-third of hepatic transcription units. It also acts as a key transcription factor for HBV cccDNA transcription through binding to HBV promoters [61,62,75]. Through surveying several key hepatic TFs regulating HBV transcription, we found that TRIM65 indeed reduces the steady-state level of the HNF4α protein in both HepG2 and Huh7 cells (Figure 5A,B). Interestingly, consistent with the mapped functional domains for the antiviral activity of TRIM65, the RING finger domain deletion mutant and the B-box deletion mutant failed to downregulate the HNF4α protein, and treatment with the proteosome inhibitor MG132 completely restored HNF4α in the presence of wild-type TRIM65 (Figure 5C), indicating that HNF4α is targeted by TRIM65 for degradation and the loss of HNF4α results in the downregulation of HBV transcription.

A previous study has reported that HNF4α binds to a specific region within the HBV enhancer I region [41]. However, TRIM65 remained active in spite of the suboptimal transcription of the HBV genome with an HNF4α binding site mutation (Figure 5D). The reason behind this is currently unclear, but possible explanations include but are not limited to (1) additional HNF4α binding sites within the EnII/Cp region [76,77,78]; (2) a reduction in other cellular TF(s) and/or factor(s) required for optimal HBV transcription due to the loss of HNF4α; (3) other HBV transcription regulatory factor(s) being downregulated by TRIM65 in parallel to TRIM65-mediated HNF4α reduction. It is worth noting that the HBV sequences in pHBV-48 and pHBV-48-4A-Mut are genotype A (Figure 5D). All the other results in this study were generated with HBV genotype D; however, the core sequences of the HNF4α binding sites are identical between genotypes A and D. Currently, we are conducting a pull-down–mass spec assay to identify TRIM65 binding proteins in HBV-transfected HepG2 cells under MG132 treatment. The proteins of interest will be assessed for their potential involvement in TRIM65-mediated ubiquitination-dependent proteosome degradation and the anti-HBV effect. 

It is well acknowledged that the major obstacle to curing chronic HBV infection is the cccDNA’s persistence in the hepatocytes, which are the authentic HBV transcription templates for HBV reproduction [79,80]. Therefore, silencing cccDNA transcription represents a promising experimental approach to developing a functional cure for HBV infection, and most likely, it will require the manipulation of host functions [5,81,82,83]. In our study, TRIM65 could inhibit the HBV EnII/Cp, SP1, and SP2 promoters to suppress viral transcription (Figure 2), and the knock-out experiment confirmed that endogenous TRIM65 is a host restriction factor in HBV infection (Figure 6), indicating that TRIM65 may be utilized to suppress cccDNA transcriptional activity by therapeutic means, pending a better understanding of its mechanism of action. 

## Figures and Tables

**Table 1:** List of 45 TRIMs and their antiviral effects on HBV replication.

	Inhibition of HBV Replication ^a^		
TRIMs	pHBV1.3 RNA Reduction	pHBV1.3 DNA Reduction	pCMVHBV RNA Reduction
1	+++	++	+
2	-	-	-
3	-	-	-
4	+++	++	-
5α	-	-	-
5δ	++++	+++	-
6	+	+	-
7	++	++++	+
8	-	+	-
9	-	+	-
10	+++	++++	+
11	+++	+++	++
13	+	++	+
14	+	++	+
15	++++	+++++	+++
18	-	-	-
19	+++	++++	+++
20	-	-	-
21	++++	+++++	++++
22	-	-	-
23	-	-	-
26	++++	++++	+++
28	-	-	-
29	-	-	-
31	++	+	+
33	+	-	-
34	+	+	-
35	+++	+	+
39	-	+	-
41	+	+	+
43	-	-	-
46	-	-	-
47	++++	+++	++
51	++	++	+
58	+++	+++	+++
64	-	-	-
65	+++++	++++	+
66	+	+	-
69	-	-	-
70	-	-	-
71	+	+	-
72	-	-	-
73	++++	+++	+++
75	-	-	-
77	-	-	-

^a^ Assays were carried out in at least duplicate; results are presented as average values. -, no reduction; +, <20% reduction; ++, 20–40% reduction; +++, 40–60% reduction; ++++, 60–80% reduction; +++++, >80% reduction.

## Data Availability

The original contributions presented in the study are included in the article, further inquiries can be directed to the corresponding authors.
